# Effect of wall slip on the viscoelastic particle ordering in a microfluidic channel

**DOI:** 10.1002/elps.202200117

**Published:** 2022-06-25

**Authors:** Gaetano D'Avino, Pier Luca Maffettone

**Affiliations:** ^1^ Dipartimento di Ingegneria Chimica dei Materiali e della Produzione Industriale Università degli Studi di Napoli Federico II Piazzale Tecchio 80 Naples 80125 Italy

**Keywords:** microfluidics, numerical simulations, particle ordering, viscoelasticity, wall slip

## Abstract

The formation of a line of equally spaced particles at the centerline of a microchannel, referred as “particle ordering,” is desired in several microfluidic applications. Recent experiments and simulations highlighted the capability of viscoelastic fluids to form a row of particles characterized by a preferential spacing. When dealing with non‐Newtonian fluids in microfluidics, the adherence condition of the liquid at the channel wall may be violated and the liquid can slip over the surface, possibly affecting the ordering efficiency. In this work, we investigate the effect of wall slip on the ordering of particles suspended in a viscoelastic liquid by numerical simulations. The dynamics of a triplet of particles in an infinite cylindrical channel is first addressed by solving the fluid and particle governing equations. The relative velocities computed for the three‐particle system are used to predict the dynamics of a train of particles flowing in a long microchannel. The distributions of the interparticle spacing evaluated at different slip coefficients, linear particle concentrations, and distances from the channel inlet show that wall slip slows down the self‐assembly mechanism. For strong slipping surfaces, no significant change of the initial microstructure is observed at low particle concentrations, whereas strings of particles in contact form at higher concentrations. The detrimental effect of wall slip on viscoelastic ordering suggests care when designing microdevices, especially in case of hydrophobic surfaces that may enhance the slipping phenomenon.

## 1 INTRODUCTION

The use of viscoelastic fluids to manipulate the particle trajectories in microfluidic applications has received a great interest in the past decade due to simpler, easy‐to‐use, and cheap devices as compared to other techniques [1, 2, 3, 4]. Fluid elasticity generates a force on the suspended particles, driving them toward specific equilibrium positions on the channel cross section. A proper selection of the fluid rheological properties can lead to a single equilibrium position coinciding with the center of the cross section, obtaining a line of particles flowing at the channel axis in a variety of cross‐section geometries and in a wide range of flow rates [1]. This “flow focusing” mechanism has been thoroughly studied for extremely dilute systems and exploited for analysis [5, 6, 7], counting [8, 9], sorting, trapping, and separation [10, 11, 12, 13, 14, 15].

As the particle concentration increases, the aligned particles can hydrodynamically interact and modify their spacing while traveling along the channel. In this regards, the formation of a single‐line equally spaced structure, referred as “particle ordering,” is desired in several applications [16], e.g., encapsulation [17, 18, 19, 20]. The first experimental evidence of the formation of self‐assembled ordered structures through fluid viscoelasticity has been recently reported [21], showing that particles suspended in an elastic and shear‐thinning liquid (i.e., the viscosity decreases with increasing the shear rate) attain a preferential spacing in a straight microchannel at high flow rates. On the contrary, elastic and constant‐viscosity fluids leads to the formation of strings of particles in contact. The capability of fluid elasticity to promote ordering has been confirmed in other types of viscoelastic liquids as well [22, 23]. An argument based on the repulsive dynamics between pairs or triplets of particles at high flow rates [24, 25] has been proposed to assess the stability of the train [21]. Numerical simulations have been used to support the experimental observations and to investigate the effect of the particle volume fraction, the distance from the channel inlet, the confinement ratio, and the polydispersity on the ordering mechanism [21, 26, 27, 28].

In all the aforementioned works, particle ordering has been investigated in water‐based polymeric solutions flowing in hydrophilic microfluidic channels where adherence condition can be assumed at walls. Similarly, the available numerical studies have considered no‐slip conditions at the channel boundaries. However, when dealing with small channels and non‐Newtonian liquids, slip at wall may occur [29, 30, 31], i.e., the fluid in contact with the channel walls has a velocity different from that of the solid surface. Wall slip depends on the nature of the liquid and the surface. Water‐based polymeric solutions may slip when in contact with hydrophobic surfaces whereas nonpolar solvents slip even at hydrophilic channel walls. The presence of slip at a liquid–solid interface modifies the fluid velocity profile along the channel, affecting, in turn, the dynamics of the suspended particles. For instance, a spherical particle suspended in a Newtonian fluid near a flat wall under creeping flow conditions experiences a drag reduction in case of a slippery surface [32]. Viscoelastic migration is also strongly affected by wall slip [33, 34]. Recent direct numerical simulations have investigated the influence of wall slip on the migration of particles in an elastic, shear‐thinning fluid flowing in a cylindrical channel [34]. In the no‐slip case, the migration direction is toward the centerline for particles released in a wide circular region of the cross section around the channel axis. On the other hand, particles near the wall migrate toward the solid surface, so that two stable equilibrium positions exist. In the presence of wall slip, the migration velocity progressively reduces as the slip length increases. At sufficiently high values of the slip length, the migration direction of a particle close to the wall reverts direction and the wall becomes an unstable equilibrium position. Hence, the particle migrates toward the channel centerline regardless of its initial position [34].

Similarly to viscoelastic particle migration, wall slip can affect particle ordering as well. Recent experiments of a suspension of spheres in xanthan gum 0.1 wt% flowing in a microchannel with hydrophobic surfaces showed the formation of strings of particles without ordering [20]. The same suspension in an hydrophilic microchannel at the same flow conditions and channel geometry self‐organized in a nearly equally spaced structure [23], remarking the relevant effect of the channel surface properties on the interparticle hydrodynamic interactions. This different behavior between hydrophilic and hydrophobic channels has been attributed to the existence of wall slip in the latter case and the consequent change of the velocity and stress profiles around the particles. Although wall slip seems to have a relevant influence on viscoelastic ordering, a systematic study on its effect on the evolution of a particle train is missing.

In this work, we investigate the dynamics of a train of particles aligned at the centerline of a cylindrical microchannel and suspended in a viscoelastic fluid in the presence of slip at channel wall. The study is carried out through numerical simulations. The Navier slip boundary condition is employed at the channel wall [32], assuming that the traction acting tangentially to the surface is proportional to the tangential component of the fluid velocity at the boundary through a “slip coefficient”. The dynamics of a system made of three aligned particles in an infinite channel is first addressed. The results are then used to simulate the multiparticle system. The evolution of the microstructure, with a special focus on particle ordering, is investigated by varying the slip coefficient, the particle concentration, and the distance from the channel inlet.

## 2 MATHEMATICAL MODEL AND SIMULATION PROCEDURE

**FIGURE 1:**
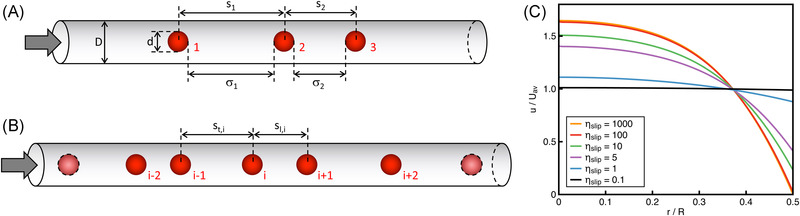
Schematic representation of the three‐particle (A) and multiparticle (B) systems. The flow goes from left to right so the particles labeled as “1,” “2,” and “3” are the trailing, middle, and leading particles, respectively. The center‐to‐center and surface‐to‐surface distances between two consecutive particles are denoted by *s* and σ . (C) Radial velocity profile of a Giesekus fluid without particles for different values of the slip parameter $\eta_{\text{slip}}$. The fluid velocity and radial position are made dimensionless by the average velocity $U_{\text{av}}$ and the channel radius *R*

The simulations of the train dynamics are carried out by employing the methodology used in our previous works [21, 26]. We consider a cylindrical channel with length *L* and diameter *D* filled by a suspension of spherical particles with diameter *d* in a viscoelastic fluid. A flow rate *Q* is imposed at the channel inlet. We assume that all the particles are aligned at the channel centerline due to the migration phenomenon induced by fluid viscoelasticity [1]. We also assume that each particle hydrodynamically interacts only with the previous and next particle of the train so that the multiparticle system can be decomposed in a set of several three‐particle systems [26]. To evolve the axial particle positions, the velocities of the particles forming the train are needed. Due to the previous assumption, with reference to Figure 1B, the velocity of a generic particle *i* only depends on the relative distance with the trailing (i−1) and leading (i+1) ones, denoted by $s_{\text{t,i}}$ and $s_{\text{l,i}}$, respectively. The calculation of these velocities is done by solving the full fluid dynamics of a system made of three particles, schematized in Figure 1A. We denote with *s*_1_ and *s*_2_ the center‐to‐center distances between the trailing and middle particles, and between the middle and trailing particles, respectively. We also consider the corresponding surface‐to‐surface distances defined as $\sigma_{1}=s_{1}-d$ and $\sigma_{2}=s_{2}-d$. To compute the velocities of the three‐particle system, the governing equations for the fluid and the particle motion need to be solved. They are the continuity and momentum balance equations for the fluid and the force balance for the particle. We assume inertialess and force‐free conditions. The viscoelastic liquid is modelled by the Giesekus constitutive equation [35]. This model well describes the rheology of several polymer‐water solutions commonly employed in microfluidics applications [21]. Regarding the boundary conditions, a flow rate *Q* is imposed at the channel inlet, periodicity is applied between the inlet and outlet channel sections, no‐slip condition is set at the particle surfaces, resulting in the rigid‐body motion condition. At the channel wall, a Navier slip condition is applied whereby the wall shear stress $\sigma_{\text{rz}}$ is proportional to the velocity of the fluid at the wall $u_{z}$, i.e., $\sigma_{\text{rz}}=-\eta_{\text{slip}}u_{z}$ with $\eta_{\text{slip}}$ a proportionality constant referred as “slip coefficient.” The limiting behaviors are obtained for $\eta_{\text{slip}}\to \infty$ and $\eta_{\text{slip}}=0$ corresponding to no‐slip and perfect slip conditions, respectively. The governing equations and boundary conditions are reported in the Supporting Information.

In this work, we fix the following dimensionless parameters: De=2, $\eta_{r}=0.1$, α=0.2, β=0.4, where $De=4\lambda Q/(\pi D^{3})$ is the Deborah number defined as the ratio between the fluid relaxation time λ and the flow characteristic time, $\eta_{r}=\eta_{s}/\eta_{p}$ is the ratio between the (Newtonian) solvent and the polymer viscosity, α is a constitutive parameter of the Giesekus model, and β=d/D is the confinement ratio. The chosen value for α gives a shear‐thinning viscosity and non‐zero normal stress differences in shear flow. These values of the parameters are chosen based on previous simulations on particle ordering where no‐slip conditions are applied at the channel wall, so an immediate comparison with the present results is possible. Figure 1C shows the radial velocity profile for a Giesekus fluid (without particles) at different values of $\eta_{\text{slip}}$. It can be readily observed that the profile at $\eta_{\text{slip}}=100$ overlaps the one at $\eta_{\text{slip}}=1000$ corresponding to the no‐slip case. (The velocity profile is flat at the channel centerline due to the fluid shear‐thinning.) As $\eta_{\text{slip}}$ reduces, the velocity profile becomes flatter up to reaching a nearly constant value at $\eta_{\text{slip}}=0.1$.

Several simulations of the three‐particle system are run by varying the two relative distances *s*_1_ and *s*_2_. Starting from a stress‐free condition, the simulations are run up to a time sufficient for the viscoelastic stresses to fully develop. Due to the very small relative particle velocities, the particle positions only slightly change during the simulation. The relative distances between the particles and the corresponding velocities obtained at the end of the simulations are stored in a look‐up table used for the train dynamics [26]. Specifically, the simulation of the multiparticle system is done by integrating the motion equation $dz_{i}/dt=V_{i}\left(s_{t,i},s_{l,i}\right)$ where $z_{i}$ and $V_{i}$ are the axial coordinate and velocity of the i−th particle. As previously discussed, $V_{i}$ only depends on the distances $s_{t,i}$ and $s_{l,i}$. The velocities are obtained by interpolating the data of the look‐up table built from the three‐particle system. This procedure allows to track in a reasonable computational time the dynamics of thousands of particles moving in a microchannel with dimensions $L/D=O(10^{3})$, commonly employed in microfluidic applications. More details on the adopted numerical method, and the simulation procedure can be found elsewhere [26].

## 3 RESULTS AND DISCUSSION

### 3.1 Three‐particle system

**FIGURE 2:**
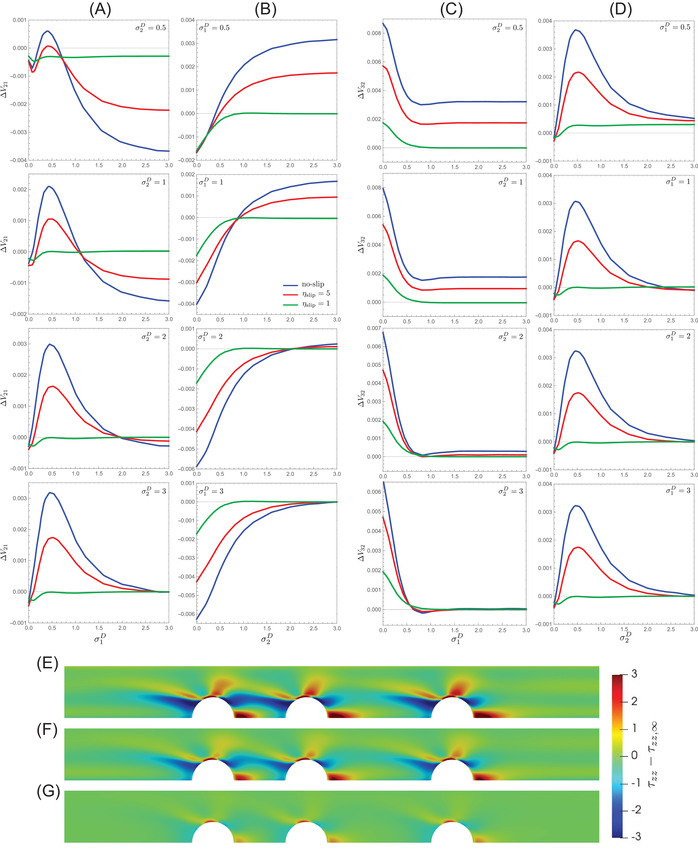
(A) Relative velocity between the middle and trailing particles as a function of the distance between the middle and leading particle surfaces $\sigma_{1}^{D}$ normalized by the channel diameter. Each panel refers to a fixed distance between the leading and middle particle surfaces $\sigma_{2}^{D}$, increasing from top to bottom. In each plot, curves for different slip parameter $\eta_{\text{slip}}$ are reported. (B) The same as in (A) with $\sigma_{2}^{D}$ reported on the *x*‐axis and for fixed values of $\sigma_{1}^{D}$. (C,D) The same as in (A) and (B) with the relative velocity between the leading and middle particles plotted on the *y*‐axis. (E–G) Color maps of the difference between the zz−component of the (dimensionless) viscoelastic stress tensor $\tau_{zz}$ and the same component of the unperturbed fluid $\tau_{zz,\infty }$ for (E) no‐slip case, (F) $\eta_{\text{slip}}=5$, (G) $\eta_{\text{slip}}=1$. The interparticle distances are $\sigma_{1}^{D}=0.5$ and $\sigma_{2}^{D}=1$

The results for the three‐particle system are first presented as they give useful information about the effect of the wall slip on the multiparticle dynamics. Figure 2A–D reports the relative particle velocities as a function of the interparticle distances for the no‐slip case (green curves) and two values of the slip coefficient $\eta_{\text{slip}}=5$ (red curves) and $\eta_{\text{slip}}=1$ (green curves). The relative velocity between the middle and the trailing particles is denoted by $\Delta V_{21}=V_{2}-V_{1}$ and the relative velocity between the leading and the middle particles is denoted by $\Delta V_{32}=V_{3}-V_{2}$. Negative or positive values of these velocities correspond to approaching or separating particles. The interparticle distances $\sigma_{1}^{D}$ and $\sigma_{2}^{D}$ used in these plots are the distances between the particle surfaces (σ_1_ and σ_2_ in Figure 1A) made dimensionless by the channel diameter. A value of $\sigma^{D}=0$ corresponds to two particles in contact. Figure 2A shows the relative velocity between the middle and the trailing particles by varying the distance between these two particles for four fixed values of the distance between the leading and middle particle (increasing from top to bottom). The panels in Figure 2B report the same quantity by fixing now the the distance between the trailing and middle particles and as a function of the other distance. In the panels in Figure 2C,D the relative velocity between the leading and middle particles is shown for the same distance values and ranges of the left panels. A remarkable quantitative effect of the wall slip can be readily observed. As the slip coefficient decreases, the curves move towards the *x*‐axis of the plots, i.e., the magnitude of the relative velocities decrease, regardless of the values of the interparticle distances. In terms of particle dynamics, this effect implies that the variations of the particle spacing are much slower as the wall slip increases, which is clearly detrimental for particle ordering. For a moderate wall slip ($\eta_{\text{slip}}=5$, red curves), the trends are qualitatively similar to the no‐slip case. Also, the critical distances at which the relative velocities change sign (from repulsive to attractive or vice versa) do not significantly change. Hence, from the no‐slip to the case at $\eta_{\text{slip}}=5$, the microstructure should evolve following a similar dynamics but over an axial distance from the channel inlet higher for the slipping case. On the contrary, a significant change is observed for the lowest value of the slip coefficient considered ($\eta_{\text{slip}}=1$, green curves). In this case, a non‐zero relative particle velocity is observed only for particles very close one to each other, i.e., for $\sigma^{D}<0.5$ corresponding to a surface‐to‐surface distance of 1.25 particle diameters. As a consequence, we expect that, for a relevant slip at the channel wall and at low particle concentrations (where the average particle spacing is large), the train travels along the microchannel without significantly changing its microstructure.

To relate the wall slip to the reduction of the relative particle velocities, we inspect the stress fields in the fluid around the particles. Figure 2E–G shows the zz−component (with *z* the axial direction) of the viscoelastic stress tensor $\tau_{zz}$ minus the same component of the fluid without particles. This quantity gives the perturbation of the axial viscoelastic normal stress due to the presence of the particles and has been previously considered to explain the attractive or repulsive motion of a particle pair [25]. The interparticle distances considered in the figure are $\sigma_{1}^{D}=0.5$ and $\sigma_{2}^{D}=1$, corresponding to relevant quantitative differences between the particle relative velocities as the slip coefficient is varied as visible in the second panels of Figure 2A and 2C at $\sigma_{1}^{D}=0.5$. The stress field distributions around the particles are qualitatively similar as the slip is varied, with a high stress region (in red) upstream of each particle and in the gap between the particles and the wall, interspersed with two low stress regions (in blue). For the particle pairs, the attracting/separating dynamics was attributed to the imbalance between the normal stresses around the particles [25]. Specifically, as the interparticle spacing reduces, the normal stress is more uniform in the gap as the fluid between the two particles travels at approximately the same velocity of the pair, leading to a small velocity gradient that, in turn, produces low viscoelastic stresses. In this case, the stress gradient between the particles is not able to contrast the external stresses resulting in particles attraction. The snapshots in Figure 2 show that the intensity of the colors reduces as the slip is enhanced. This is strictly connected with the flatter velocity profile displayed in Figure 1C and the consequent reduction of the shear rate around the particles that, in turn, reduces the stress magnitude. Hence, although the relative particle motion does not qualitatively change (attractive or repulsive), the magnitude of the net force that generates such a motion reduces as the wall slip increases. For the most slipping case investigated in this work (Figure 2G), the perturbation of the stress field extends for a small region around the particles, implying a weak effect of the hydrodynamic interactions. Indeed, the stress field distributions around the three particles in Figure 2G are very similar, i.e., the particles behave like isolated objects, leading to a (almost) zero relative velocity and no significant variation of their spacing.

### 3.2 Multiparticle system

The results of the three‐particle system are used to investigate the dynamics of a train of particles in a long cylindrical microchannel. The analysis is carried out by varying the particle volume fraction and the slip coefficient, and the results are presented at different distances from the channel inlet. Since all the particles are aligned at the channel centerline, as a measure of the volume fraction we consider the linear concentration defined as the length of the channel centerline occupied by the particles divided by the total channel length [36]:

(1) $$\phi =\frac{Nd}{L}$$

where *N* is the number of particles flowing in the channel with length *L*. The investigated range of the linear concentration goes from ϕ=0.1 to ϕ=0.4, corresponding to an average surface‐to‐surface spacing of 9 and 1.5 particle diameters, respectively. Following our previous work [26], we normalized the interparticle spacing as follows:

(2) $$\sigma_{\text{norm}}=\frac{s-1}{2(s_{0}-1)}=\frac{\sigma }{2\sigma_{0}}$$

where *s*_0_ and σ_0_ are the center‐to‐center and the surface‐to‐surface average distances at the channel inlet. In this way, the initial distances are uniformly distributed between 0 and 1 regardless of the linear concentration. The interparticle spacing distributions are evaluated at different distances from the channel inlet made dimensionless with the channel diameter, $\Lambda =Z_{\text{obs}}/D$, where $Z_{\text{obs}}$ is the axial distance from the inlet at which the interparticle spacing is monitored. We consider a range of Λ from 500 to 3000. Just to give an idea, for a channel with a diameter of 100 microns, a value of Λ=3000 corresponds to a distance from the inlet of 30 centimeters. It is worth pointing out that by “inlet” we intend the section of the channel at which the particles are released in the computational domain. As previously discussed, in a real microfluidic device, such a section corresponds to a certain distance from the channel feeding section at which all the particles have migrated at the centerline.

**FIGURE 3:**
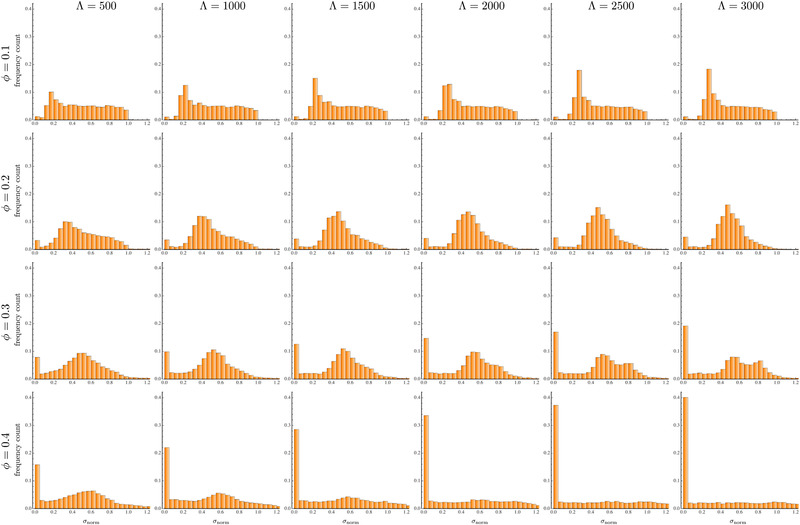
Histograms of the interparticle distance distributions for different (dimensionless) distances from the channel inlet Λ (increasing from left to right) and linear concentration ϕ (increasing from top to bottom) for a no‐slip boundary condition at the channel wall. The axis of the plots is the dimensionless distance normalized according to Equation (2)

The spacing distributions for the no‐slip case at different linear concentrations (increasing from top to bottom) and distances from the channel inlet (increasing from left to right) are shown in Figure 3. A direct quantitative comparison with previous simulations [26] is not possible due the different definition of the particle concentration (the linear concentration is used in the present work whereas the volume fraction was considered in Ref. [26]). Furthermore, the simulations by D'Avino and Maffettone were limited to Λ=1000 whereas now a total channel length three times higher is considered. However, qualitative similarities with the results reported in Ref. [26] can be observed. At the lowest particle concentration (ϕ=0.1), a preferential spacing at $\sigma_{\text{norm}}\approx 0.25-0.3$ is visible, corresponding to a surface‐to‐surface distance of about 4.5–5 diameters. As expected, the particles that tend to order are those at initial distances smaller than the equilibrium one, for which the hydrodynamic interactions are stronger (the bars at $\sigma_{\text{norm}}<0.2$ become shorter as the distance from the channel inlet increases). For the opposite reason, particles at large initial distances ($\sigma_{\text{norm}}>0.4$) are essentially isolated and keep their distance till the channel end. At ϕ=0.2, a preferential spacing is still observed at $\sigma_{\text{norm}}\approx 0.5$, corresponding to a surface‐to‐surface distance of about 4 diameters. Although the average distance is similar to the case at ϕ=0.1, the distributions show that all the particles, regardless of the initial distance, tend to form an ordered structure (the tail of the distribution at ϕ=0.2 and Λ=3000 is clearly lower than the one at Λ=500). This is due to the shorter average interparticle distance that enhances hydrodynamic interactions as compared to the case at ϕ=0.1. The distributions also show the appearance of a bar at $\sigma_{\text{norm}}\approx 0$ denoting the formation of strings of particles in contact. Such a bar is, however, relatively low so that only a small fraction of particles (about 6%) form strings. By increasing the linear concentration to ϕ=0.3, the distribution switches from uni‐modal to bi‐modal at Λ=3000, with a preferential spacing at $\sigma_{\text{norm}}\approx 0.5$ and a second peak at $\sigma_{\text{norm}}\approx 0.8$. More importantly, the leftmost bar increases with increasing the distance from the channel inlet, denoting that more and more particles are arranging in strings. The formation of strings of particles in contact is even more evident at the largest linear concentration investigated (ϕ=0.4) where no ordering is observed. As a final comment, notice that, for all the concentrations, a steady‐state distribution is never attained at the largest distance from the inlet, confirming that viscoelastic ordering is a slow process [16, 23].

**FIGURE 4:**
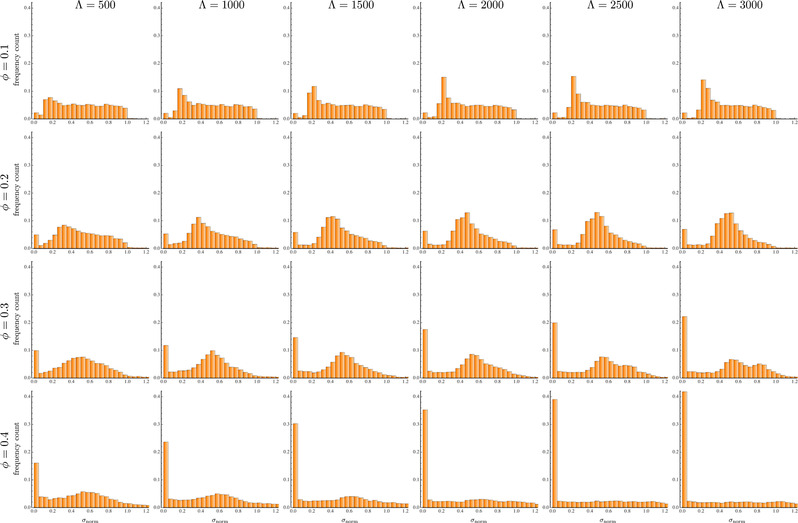
Histograms of the interparticle distance distributions for different (dimensionless) distances from the channel inlet Λ (increasing from left to right) and linear concentration ϕ (increasing from top to bottom) for a slip boundary condition at the channel wall with $\eta_{\text{slip}}=5$. The axis of the plots is the dimensionless distance normalized according to Equation (2)

The same histograms are reported in Figure 4 for a slip coefficient $\eta_{\text{slip}}=5$. A comparison with the plots in Figure 3 shows that: i) the distributions are qualitatively similar, with a preferential distance at ϕ=0.1 and ϕ=0.2, a bi‐modal distribution at ϕ=0.3 and Λ=3000 with several particles in contact, and a very large amount of particles forming strings at ϕ=0.4, ii) the values of $\sigma_{\text{norm}}$ corresponding to the preferential spacing are also similar between the two cases, iii) at ϕ=0.1 and ϕ=0.2, the evolution of the distributions is slower for the slipping case; for instance, the histograms at $\eta_{\text{slip}}=5$ and Λ=3000 are similar to the no‐slip case at Λ=1500÷2000. This difference is a consequence of the smaller relative velocities discussed in the previous section. As shown in Figure 2, the relative velocities between the three particles for $\eta_{\text{slip}}=5$ (red curves) show the same trend of the no‐slip case (blue curves) with a vertical scaling factor of about 2. Such a scaling works well for surface‐to‐surface interparticle distances higher than 1÷1.5 diameters, corresponding to about $\sigma^{D}>0.5$. Hence, at low linear concentrations, the spacing is rather large and the relative velocity scaling applies, confirming that a very similar distribution for $\eta_{\text{slip}}=5$ is found at a distance from the inlet that is twice the value for the no‐slip case. As the linear concentration increases, the system is more crowded, the particles get closer, and the scaling is lost. In particular, the curves in Figure 2 at $\sigma^{D}<0.5$ show that the relative velocity magnitude for the slipping case is comparable or even larger than the no‐slip case. Consequently, the probability to form particle strings is higher, as visible from the histograms in Figure 4 for ϕ=0.3 and ϕ=0.4.

**FIGURE 5:**
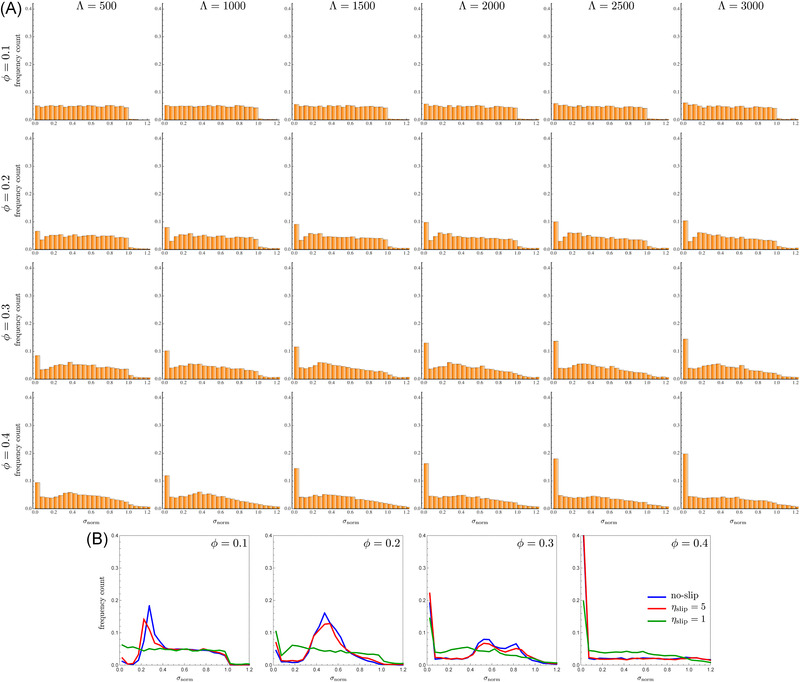
(A) Histograms of the interparticle distance distributions for different (dimensionless) distances from the channel inlet Λ (increasing from left to right) and linear concentration ϕ (increasing from top to bottom) for a slip boundary condition at the channel wall with $\eta_{\text{slip}}=1$. The axis of the plots is the dimensionless distance normalized according to Equation (2). (B) Comparison between the interparticle distributions for different values of the slip coefficient and linear concentration at Λ=3000

Figure 5A reports the spacing distributions for a slip coefficient $\eta_{\text{slip}}=1$. A drastic change from the previous cases is observed. At ϕ=0.1, the spacing distribution does not change in the whole channel length. Indeed, the distributions remain essentially uniform even at Λ=3000, i.e., the same initial (random) microstructure is found at the channel exit. As the linear concentration increases, we observe an increasing probability to form particle strings without the appearance of a preferential interparticle distance. This behavior can be again explained by considering the three‐particle system results. The green curves in Figure 2 shows that, at $\sigma^{D}>0.5$, the relative velocities are roughly zero. At a linear concentration of ϕ=0.1, about 7% of the particles are at a surface‐to‐surface distance $\sigma^{D}$ lower the 0.5 and, indeed, the relative particle positions remain unchanged except for a small increase of the leftmost bar of the histograms. The fraction of particles at a distance $\sigma^{D}<0.5$ increases at about 15%, 27%, and 42% for ϕ=0.2, ϕ=0.3, and ϕ=0.4, respectively, justifying the progressive increasing of particle strings, without significant changes of the spacing between the particles at distances $\sigma^{D}>0.5$. A comparison of the interparticle distributions for different slip coefficients and linear concentration at Λ=3000 are shown in Figure 5B. The curves denote the height of the bars shown in the previous plots. At $\eta_{\text{slip}}=5$, the distributions are only slightly affected as compared to the no‐slip case. On the other hand, the detrimental effect of a strong wall slip is readily visible at low/moderate linear concentrations.

In summary, a moderate slip at wall makes the ordering still possible although the process becomes slower and a longer channel is needed. A strong wall slip prevents any ordering mechanism and, at sufficiently large linear concentrations, only strings of particles in contact are formed. These simulation results are in line with recent experiments where a suspension of spherical particles in a shear‐thinning xantham gum 0.1 wt% in a hydrophobic microchannel was considered [20]. As discussed in the Introduction, in contrast with previous experiments of the same suspension in a hydrophilic channel [23], no ordering was observed and the particles formed aggregates. The present results confirm what postulated by the authors that a modification of the surface properties from hydrophilic to hydrophobic leads to a variation of the shape of the normal stress profiles, reducing the attraction/repulsion driving force.

## 4 CONCLUDING REMARKS

The dynamics of a particle train in a viscoelastic fluid flowing in a microchannel in the presence of wall slip is investigated by numerical simulations. A system made of three particles in an infinite channel is first analyzed by direct numerical simulations. For a moderate wall slip, the relative velocities between the particles are lower than the no‐slip case, although the trends as function of the interparticle distances are qualitatively similar. As the slip coefficient is further decreased, the relative velocities become roughly zero for surface‐to‐surface particle distances higher than 1–1.5 diameters, being however small in magnitude for closer particles. The magnitude of the axial component of the fluid viscoelastic stress (relative to the unperturbed field) reduces for decreasing slip coefficients. For the most slipping case, the perturbation of the stress field extends to a small region around the particles, implying a weak effect of the hydrodynamic interactions, and a consequent small velocity difference between the particles.

The results of the three‐particle system are used to investigate the multiparticle dynamics. The basic assumption is that each particle hydrodynamically interacts only with the trailing and leading one. Hence, the velocity of each particle of the train is taken by interpolating the direct numerical simulation results of the three‐particle case. The histograms of the particle spacing are analyzed by varying the slip coefficient, the linear concentration, and the distance from the channel inlet. For the no‐slip and moderate slip cases, a preferential particle spacing is observed for low/medium linear concentrations. The distributions of the slipping case at some distance from the channel inlet are similar to the no‐slip one at shorter distances from the inlet, so the wall slip slows down the ordering dynamics. For a strong fluid‐wall slip, ordering is completely suppressed. At low particle concentrations, the spacing distribution remains uniform at any investigated distance from the channel inlet. At higher concentrations, the particles arrange in strings.

The present results elucidate the relevant and detrimental effect of wall slip on the viscoelastic ordering efficiency, suggesting to properly evaluate the fluid behavior near the channel wall to design microdevices, especially in case of water‐based polymeric solutions in contact with hydrophobic surfaces or nonpolar solvents at hydrophilic walls where slip might be relevant.

## CONFLICT OF INTEREST

The author has declared no conflict of interest.

## Supporting information


Supporting Information

## Data Availability

The data that support the findings of this study are available from the corresponding author upon reasonable request.
