# Supplementary material for: Effect of wall slip on the viscoelastic particle ordering in a microfluidic channel
Source: Electrophoresis. 2022 Jun 25;43(21-22):2206–16. doi: 10.1002/elps.202200117 (PMC9796797; doi:10.1002/elps.202200117)
Supplement: Supplementary file 1 — Supporting Information [file ELPS-43-2206-s001.pdf]

# Supporting information for 'Effect of wall slip on the viscoelastic particle ordering in a microfluidic channel'

Gaetano D'Avino, Pier Luca Maffettone

Dipartimento di Ingegneria Chimica,

dei Materiali e della Produzione Industriale

Università degli Studi di Napoli Federico II

Piazzale Tecchio 80, 80125 - Naples, Italy

gaetano.davino@unina.it; pierluca.maffettone@unina.it

## S1 Governing equations

The dynamics of the three-particle system is computed through Direct Numerical Simulations, i.e., by solving the fluid and particle governing equations. Assuming incompressibility and negligible inertia for both solid and fluid, the governing equations are the mass and momentum balance equations:

$$\nabla \cdot \boldsymbol{u} = 0 \tag{1}$$

$$\nabla \cdot \boldsymbol{\sigma} = \mathbf{0} \tag{2}$$

where $\boldsymbol{u}$ is the fluid velocity and $\boldsymbol{\sigma}$ is the total stress tensor, expressed as:

$$\boldsymbol{\sigma} = -p\boldsymbol{I} + 2\eta_{\mathrm{s}}\boldsymbol{D} + \boldsymbol{\tau} \tag{3}$$

In Eq. (3), $p$, $\boldsymbol{I}$, $\eta_\mathrm{s}$, $\boldsymbol{D} = (\nabla\boldsymbol{u} + (\nabla\boldsymbol{u})^T)/2$, and $\boldsymbol{\tau}$ are the pressure, the unity tensor, the viscosity of a Newtonian 'solvent', the rate-of-deformation tensor, and the viscoelastic stress, respectively. The Giesekus model is chosen as constitutive equation [32]:

$$\lambda \overset{\nabla}{\boldsymbol{\tau}} + \frac{\alpha\lambda}{\eta}\boldsymbol{\tau}\cdot\boldsymbol{\tau} + \boldsymbol{\tau} = 2\eta_\mathrm{p}\boldsymbol{D} \tag{4}$$

where $\eta_\mathrm{p}$ is the polymer viscosity, $\lambda$ is the fluid relaxation time, the symbol $(^{\nabla})$ denotes the upper-convected time derivative:

$$\overset{\nabla}{\boldsymbol{\tau}} \equiv \frac{\partial\boldsymbol{\tau}}{\partial t} + \boldsymbol{u}\cdot\nabla\boldsymbol{\tau} - (\nabla\boldsymbol{u})^T\cdot\boldsymbol{\tau} - \boldsymbol{\tau}\cdot\nabla\boldsymbol{u} \tag{5}$$

and $\alpha$ is a constitutive parameter. The zero-shear viscosity is defined as $\eta_0 = \eta_\mathrm{s} + \eta_\mathrm{p}$. For $\alpha = 0$, the model recovers the Oldroyd-B constitutive equation. For positive values of this parameter, the model predicts (in shear) shear-thinning for the viscosity and for both the first and second normal stress difference coefficients $\Psi_1 = N_1/\dot{\gamma}^2$ and $\Psi_2 = N_2/\dot{\gamma}^2$ where $N_1$ and $N_2$ are the first and second normal stress differences.

# S2 Boundary conditions

Since the particles are aligned at the centerline, we can reduce the 3D domain to a 2D axisymmetric one and consider cylindrical coordinates $(r, z)$. No-slip and rigid-body motion are imposed at the sphere surfaces:

$$\boldsymbol{u} = (u_\mathrm{r}, u_\mathrm{z}) = (0, V_i) \tag{6}$$

where $V_\mathrm{i}$ is the particle axial translational velocity and $i = [1, 2, 3]$ is the particle number. The Navier slip condition is set at the channel wall:

$$(\boldsymbol{I} - \boldsymbol{nn})\cdot(\boldsymbol{\sigma}\cdot\boldsymbol{n}) = -\eta_\mathrm{slip}(\boldsymbol{I} - \boldsymbol{nn})\cdot\boldsymbol{u} \tag{7}$$

where $\boldsymbol{I} - \boldsymbol{nn}$ is the tangential projection operator, $\boldsymbol{n}$ is the normal vector at the surface, and $\eta_{\text{slip}}$ a proportionality constant referred as 'slip coefficient'. For a cylinder, the above equation reduces to $\sigma_{\text{rz}} = -\eta_{\text{slip}} u_{\text{z}}$ with $\sigma_{\text{rz}}$ the wall shear stress.

Axial symmetry is applied on the boundaries representing the axis of symmetry:

$$\boldsymbol{u} \cdot \hat{\boldsymbol{r}} = 0 \tag{8}$$

$$(\boldsymbol{\sigma} \cdot \hat{\boldsymbol{r}})\,|_z = 0 \tag{9}$$

where $\hat{\boldsymbol{r}}$ is the unit vector along the radial direction.

Periodic boundary conditions are prescribed between the inflow and outflow sections, together with a flow rate in inflow:

$$\boldsymbol{u}|_{\partial\Omega_{\text{in}}} = \boldsymbol{u}|_{\partial\Omega_{\text{out}}} \tag{10}$$

$$(\boldsymbol{\sigma} \cdot \hat{\boldsymbol{z}})\,|_{\partial\Omega_{\text{in}}} = (\boldsymbol{\sigma} \cdot \hat{\boldsymbol{z}})\,|_{\partial\Omega_{\text{out}}} - \Delta p\,\hat{\boldsymbol{z}} \tag{11}$$

$$\int_{\partial\Omega_{\text{in}}} \boldsymbol{u} \cdot \hat{\boldsymbol{z}}\, dS = Q \tag{12}$$

where $\hat{\boldsymbol{z}}$ is the unit vector along the $z-$direction and $\Delta p$ is the pressure drop along the channel between the inflow $\partial\Omega_{\text{in}}$ and outflow $\partial\Omega_{\text{out}}$ sections. The flow rate $Q$ is imposed through a constraint where the associated Lagrange multiplier is identified as the unknown pressure difference $\Delta p$. Due to the periodicity along the $z-$direction, the domain length $L$ must be chosen much larger than the channel diameter in order to avoid that the particles can hydrodynamically interact with their images. We found that a length of $L = 40d$ is sufficient to satisfy this condition.

# References

[1] D'Avino, G., Greco, F., Maffettone, P. L, *Annu. Rev. Fluid Mech.* 2017, *49*, 341-360.

[2] Yuan, D., Zhao, Q., Yan, S., Tang, S.-Y., Alici, G., Zhang, J., Li, W., *Lab Chip* 2018, *18*, 551-567.

[3] Manshadi, M. K. D., Mohammadi, M., Monfared, L. K., Sanati-Nezhad, A., *Biotechnol. Bioeng.* 2019 , *117*, 580-592.

[4] Zhou, J., Papautsky, I., *Microsystems & Nanoengineering* 2020, *6*, 113.

[5] Dannhauser, D., Romeo, G., Causa, F., De Santo, I., Netti, P. A., *Analyst* 2014, *139*, 5239-5246.

[6] Dannhauser, D., Maremonti, M. I., Panzetta, V., Rossi, D., Netti, P. A., Causa, F., *Lab Chip* 2020, *20*, 4611-4622.

[7] Serhatlioglu, M., Asghari, M., Tahsin Guler, M., Elbuken, C., *Electrophoresis* 2019, *40*, 906-913.

[8] Lee, Y., Kim, B., Choi., S., *Sens. Actuator A Phys.* 2020, *309*, 112038.

[9] Liu, C., Xue, C., Chen, X., Shan, L., Tian, Y., Hu, G., *Anal. Chem.* 2015, *87*, 60416048.

[10] Li, D., Lu, X., Xuan, X., *Anal. Chem.* 2016, *88*, 1230312309.

[11] Zhou, Y., Ma, Z., Ai, Y., *Lab Chip* 2020, *20*, 568-581.

[12] Kwon, T., Choi, K., Han, J., *Small* 2021, *17*, 2101880.

[13] Del Giudice, F., D'Avino, G., Maffettone, P. L., *Lab Chip* 2021, *21*, 2069-2094.

[14] Edd, J. F., Di Carlo, D., Humphry, K. J., Koster, S., Irimia, D., Weitz, D. A., Toner, M., *Lab Chip* 2008, *8*, 1262-1264.

[15] Rotem, A., Ram, O., Shoresh, N., Sperling, R. A., Schnall-Levin, M., Zhang, H., Basu, A., Bernstein, B. E., Weitz, D. A., *PLoS ONE* 2015, *10*, e0116328.

[16] Li, L., Wu, P., Luo, Z., Wang, L., Ding, W., Wu, T., Chen, J., He, J., He, Y., Wang, H., Chen, Y., Li, G., Li, Z., He, L., *ACS Sens.* 2019, *4*, 12991305.

[17] Shahrivar, K., Del Giudice, F., *Soft Matter* 2021, *35*, 8068-8077.

[18] Del Giudice, F., D'Avino, G., Greco, F., Maffettone, P. L., Shen, A. Q., *Phys. Rev. Appl.* 2018, *10*, 064058.

[19] Liu, L., Xu, H., Xiu, H., Xiang, N., Ni, Z., *Analyst* 2020, *145*, 5128-5133.

[20] Jeyasountharan, A., Shahrivar, K., D'Avino, G., Del Giudice, F., *Anal Chem.* 2021, *93*, 5503-5512.

[21] D'Avino, G., Hulsen, M. A., Maffettone, P. L., *Comput. Fluids* 2013, *86*, 45-55.

[22] D'Avino, G., Maffettone, P. L., *Microfluid. Nanofluid.* 2019, *23*, 82.

[23] D'Avino, G., Maffettone, P. L., *Meccanica* 2020, *55*, 317-330.

[24] Hu, X., Lin, P., Lin, J., Zhu, Z., Yu, Z., *J. Fluid Mech.* 2022, *936*, A5.

[25] Jeyasountharan, A., D'Avino, G., Del Giudice, F., *Phys. Fluids* 2022, *34*, 042015.

[26] Tretheway, D. C., Meinhart, C. D., *Phys. Fluids* 2002, *14*, L9.

[27] Lauga, E., Brenner, M., Stone, H., Microfluidics: The No-Slip Boundary Condition. In: Tropea C., Yarin A.L., Foss J.F. (eds) *Springer Handbook of Experimental Fluid Mechanics*, Springer, Berlin, Heidelberg, 2007.

[28] Hatzikiriakos, S. G., *Soft Matter* 2015, *11*, 7851-7856.

[29] Luo, H., Pozrikidis, C., *J. Eng. Math.* 2008 , *62*, 1-21.

[30] Tehrani, M. A., *J. Rheol.* 1996, *40*, 1057-1077.

[31] Trofa, M., D'Avino, G., Hulsen, M. A., Maffettone, P. L., *J. Non-Newtonian Fluid Mech.* 2016, *236*, 123-131.

[32] Larson, R.G., *The Structure and Rheology of Complex Fluids* 1999, Oxford University Press.

[33] Kahkeshani, S., Haddadi, H., Di Carlo, D., *J. Fluid Mech.* 2015, *786*, R3.
